# Less Intensive Regimens May Still Be Suitable for the Initial Treatment of Primary Mediastinal B-Cell Lymphoma in Resource-Limited Settings

**DOI:** 10.1155/2022/2099456

**Published:** 2022-06-06

**Authors:** Rodrigo Dolphini Velasques, Wellington F da Silva, Marcelo Bellesso, Vanderson Rocha, Juliana Pereira

**Affiliations:** ^1^Instituto do Cancer do Estado de São Paulo, São Paulo, SP, Brazil; ^2^Division of Hematology, Transfusion Medicine and Cell Therapy, Hospital das Clinicas da Faculdade de Medicina da USP, Sao Paulo, SP, Brazil; ^3^Laboratory of Medical Investigation in Pathogenesis and Targeted Therapy in Onco-Immuno-Hematology (LIM-31), Department of Hematology, Hospital das Clinicas da Faculdade de Medicina da USP, Sao Paulo, SP, Brazil; ^4^Churchill Hospital, Oxford University, Oxford, UK

## Abstract

Primary mediastinal B-cell lymphoma (PMBCL) is an uncommon disease, consisting of 2–4% of non-Hodgkin lymphomas. Radiotherapy-free DA-EPOCH-R and R-CHOP plus radiotherapy (RT) have been the upfront standard regimens worldwide. However, performing DA-EPOCH-R in resource-constrained settings can be burdensome, especially in low/middle-income countries, where data on PMBCL are still largely unknown. We retrospectively analyzed 93 patients with PMBCL diagnosed between 2008 and 2018 with the intention of comparing the characteristics of the patients and the results obtained with each protocol and to verify if the use of less intensive chemotherapy is still possible to be used. The median age was 28 years, 59.1% were female, 42.3% were in advanced stages, and 92.1% were with bulky disease. DA-EPOCH-R (41.9%), R-CHOP (35.5%), and R-CHOEP (22.6%) were the regimens used, and no difference was observed in the characteristics of the patients. After four cycles of chemotherapy, complete response (CR), partial response (PR), and progressive disease (PD) rates were 40%, 55.7%, and 4.5%, respectively. At the end of treatment, metabolic CR and PD rates were 56.8% and 11.1%. RT was performed in 42.1% of DA-EPOCH-R, 75% of R-CHOP, and 83% of R-CHOEP, and switched PR to CR in 73.7%. Estimated 5-year PFS and OS were 77.2% and 77.4%, respectively. Only LDH levels remained independently associated with PFS, and type of treatment was not associated with OS, PFS, or relapse rate. Therefore, we conclude that in a resource-constrained setting, R-CHOP or R-CHOEP could be still safely adopted in upfront treatment for PMBCL.

## 1. Introduction

Primary mediastinal B-cell lymphoma (PMBCL) is an uncommon lymphoproliferative disease arising from mature thymic B-cells and comprises about 2 to 4% of all non-Hodgkin's lymphomas (NHLs) [1]. It has unique clinical, immunophenotypic, genotypic, and molecular features, closer to nodular sclerosis classic Hodgkin's lymphoma (cHL) than to diffuse large B-cell lymphoma (DBCL) itself—an entity with which it was previously related. Thus, it ended up being established as a distinct disease by the World Health Organization (WHO) classification in 2008 [2].

The clinical management of PMBCL differs between centers, but invariably consists of anthracycline-based chemotherapy with rituximab, with or without consolidative mediastinal irradiation (RT) [3]. Studies with CHOP-like regimens, selected over the years, show progression-free survival (PFS) of 69 to 88% with notable benefit from rituximab-containing regimens [4–10]. However, the benefit of RT is still uncertain [8, 11]. Although it appears to be mandatory after R-CHOP [12], this may not be true for more intensified approaches. The radiotherapy-free DA-EPOCH-R regimen achieved an exceptional 5-year event-free survival of 93% in its debut phase 2 study [13], although these remarkable results could not be replicated by other centers [11, 14]. A positron emission tomography-guided approach with 18F-fluorodeoxyglucose (F-FDG18 PET-CT) may be an alternative to prevent patients from the long-term deleterious effects associated with RT, but this issue is still under investigation [15].

In resource-limited settings such as Latin American countries, the adoption of the DA-EPOCH-R is logistically challenging due to the need for long-term central catheters for drug infusion and the availability of hospital beds for a 96-hour hospitalization (or else, pumps for home infusion of medicines). Not infrequently, the assisting medical team ends up having to adopt alternative strategies to maintain the treatment, making adaptations or even replacing treatment regimens. Furthermore, for the reasons cited, plus the preemptive use of colony-stimulating factors may limit the implementation of the DA-EPOCH-R. Therefore, there is also a lack of cost-effectiveness and quality of life studies after DA-EPOCH-R compared to R-CHOP in these settings.

As a rare entity, PMBCL studies rarely include more than 200 patients from a single center and, to the best of our knowledge, there are no large contemporary data in Latin America in this area. In this data collection, we intend to describe the clinical characteristics and results of patients with PMBCL treated at our center and compare the results of the main regimens performed over the years, determining the main factors of response and survival.

## 2. Materials and Methods

### 2.1. Study Design and Ethics Statement

This is a retrospective, single-center, chart review study carried out at the *Cancer* Institute of the State of Sao Paulo (ICESP) of the University of São Paulo (USP), in Sao Paulo, Brazil. Clinical and laboratorial data were obtained from the NHL patient database of the Discipline of Hematology, Hemotherapy and Cell Therapy. All procedures were performed in accordance with the ethical standards of the institutional research committee and the 1964 Declaration of Helsinki, or comparable ethical standards. Study data were collected and managed using REDCap electronic data capture tools hosted at the University of Sao Paulo [16].

### 2.2. Patients and Diagnosis

Patients aged 15 years or older, diagnosed with PMBCL and admitted to our institution between January/2008 and June/2018, were included. Individuals who were unable to receive curative chemotherapy due to poor clinical status or did not receive rituximab along with initial therapy were excluded from the study.

The diagnosis of PMBCL was performed according to the 2008 WHO classification criteria [17], after histopathological confirmation from a tissue biopsy. All biopsies performed at our center were centrally analyzed by the Department of Pathology of the Faculty of Medicine of the University of São Paulo according to their operational protocols, although not always by the same pathologist. All external material was obligatory submitted to internal review for diagnostic confirmation. Patients were staged according to the Ann Arbor classification (modified in Costwolds and Lugano) (2014) [18], preferably using PET-CT. When unavailable, whole-body CT scans were used and, when used, required the completion of staging with unilateral bone marrow biopsy. Central nervous system (CNS) disease was defined as the presence of neoplastic cells in the cerebrospinal fluid (by oncotic cytology or immunophenotyping), presence of cranial nerve palsy, clinical signs of spinal cord compression, or intracranial mass. For DA-EPOCH-R patients, lumbar puncture was indicated as described by Dunleavy et al. in the original protocol [13]. For R-CHOP/R-CHOEP patients, we followed our institutional guidelines (presence of renal, gonadal, or more than one noncontiguous extranodal site of disease). Bulky disease was defined by the presence of a mediastinal tumor mass with its largest diameter greater than 10 cm or greater than 1/3 of the chest wall width at any height of the chest. Patients with Ann Arbor stage III or IV were considered as advanced disease. Thrombotic events were screened only in patients with any symptoms or incidentally diagnosed at staging.

### 2.3. Definitions, Treatments, and Response Evaluation

Patients were treated according to our protocol which changed over time from R-CHOP plus RT to DA-EPOCH-R. Some patients received a modified regimen named “R-CHOEP” (consisting of R-CHOP plus etoposide 100 mg/^2^ on days 1 to 3), when there was no available hospital bed for DA-EPOCH-R infusion. The administration of a “COP” cytoreductive chemotherapy was performed in some patients with bulky diseases at the discretion of the attending physician at the time. Patients who received R-CHOP or R-CHOEP were scheduled for 6 or 8 courses at intervals of 21 days. Patients who achieved a complete response after the interim radiologic evaluation could complete the sixth cycle, while the remaining patients had to continue until the eighth cycle. DA-EPOCH-R patients in clinical complete response (CR) at the end of cycle 4 received two more cycles for a total of 6 cycles. Patients with an estimated <80% reduction in initial tumor bulk whose disease continued to regress between cycles 4 and 6 were referred to two more cycles following cycle 6 as suggested by the DA-EPOCH-R protocol [13]. CNS prophylaxis was performed with four cycles of intrathecal methotrexate (MTX) 12 mg along cycles 1 to 4, and for patients with good performance status and tolerance to the chosen chemotherapy, two cycles of intravenous methotrexate at 3 g/m^2^ were performed after upfront therapy. The regimens are detailed in the supplementary appendix.

Radiologic assessments were performed by CT or PET-CT scans at the end of cycles 4 and 6. R-CHOP/R-CHOEP patients scheduled to receive eight cycles were re-evaluated only after the 8th cycle. Assessments were categorized according to the Cheson and Lugano criteria for CT and PET-CT, respectively [18, 19]. For the subset of patients assessed computed tomography, we adopted the category of unconfirmed complete response (CRu) classification, when there is a decrease of at least 75% of the products of the two largest diameters of the remaining mediastinal mass. Those who showed only stable disease (SD) or progressive disease (PD) at any stage of assessment were considered primary refractory and referred to salvage therapy.

**Figure 1:**
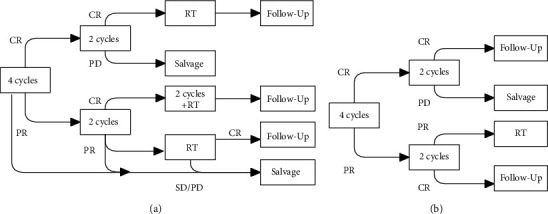
Flowchart of treatment according to regimens adopted: (a) R-CHOP and R-CHOEP; (b) DA-EPOCH-R.

Although consolidative RT was advised in all R-CHOP and R-CHOEP patients at the end of chemotherapy, it was also given to DA-EPOCH-R subjects who did not achieve CR at the end of treatment. Recommendations for regimen management are shown in Figure 1.

### 2.4. Statistical Analysis

Pairwise comparisons between patient subgroups were performed using the Mann–Whitney or Kruskal–Wallis tests for continuous variables and by Pearson's chi-square or Fisher's exact test for categorical variables. Progression-free survival was calculated from the start of treatment to the date of no response, relapse, or death. Overall survival was calculated from the date of diagnosis until death. Survival curves were drawn using the Kaplan–Meier method and compared using log-rank test. The mean follow-up time was estimated by reversing the censoring indicator codes in the Kaplan–Meier analysis. The cumulative incidence of relapse (CIR) was calculated considering death as a competitor and compared using Grey's test [20]. The association between the factors was investigated by logistic regression and, the factors associated with survival outcomes, by Cox regression. Univariate (UVA) risk estimates were generated with unadjusted Cox proportional hazard models. Covariates demonstrating significance of *p* < 0.10 in the univariate analysis were included in the multivariate (MVA) model. These models were compared to find the most accurate using the corrected Akaike information criterion (cAIC) [21]. All analyses were done using *R* software package version v 3.5.1 (*R* Foundation for Statistical Computing; https://www.r-project.org), and a two-sided *p*-value <0.05 was considered statistically significant.

## 3. Results

### 3.1. Patients' Characteristics

Initially, 95 patients were evaluated, while two were excluded from analyses, as they could not undergo any treatment due to low performance status (Table 1). The median age at diagnosis was 28 years (range, 15–74 years), and 55 (59.1%) were female. PET-CT was the staging modality for 85% of cases. Just under half of the patients were classified as having advanced disease, while 32% had an extranodal presentation (stage IV). Most patients (92%) were diagnosed with bulky disease, and none had CNS involvement in staging. Lactate dehydrogenase (LDH) was above than upper limit of normal (ULN ranging from 214 to 480 U/L, according to laboratory reference at the time of sampling) in 87% of patients.

Thrombosis was detected in 33 patients (37%), predominantly in cervical and mediastinal veins (29/33, 88%), followed by lower extremities (2, 6.1%), upper extremities (2/33, 6.1%), and splanchnic veins (1/33, 3.0%). Only 9% of them (3/33) were catheter related. The presence of thrombosis, however, did not correlate with any of the variables, such as age, LDH, stage, albumin, bulky disease, blood counts, lymphocyte-monocyte ratio (LMR), smoking, or body mass index (BMI).

In UVA, laboratory parameters associated with advanced stage were the lower LMR (1.3 vs. 1.99, *p*=0.042), albumin level below the reference value (3.7 vs. 4.03, *p*=0.033), and ULN LDH level (3.3 vs. 2.1, *p*=0.02). Other variables such as BMI (*p*=0.399), smoking (*p*=0.845), bulky disease (*p*=0.130), B-symptoms (*p*=0.834), thrombosis (*p*=0.678), sex (*p*=0.443), blood counts (*p*=0.312), or age (*p*=0.698) did not reach statistical significance. In the MVA, including LMR, albumin, and LDH, only the latter remained statistically associated with advanced stages (adjusted odds ratio (OR) = 1.37 (95% CI: 1.08–1.81), *p*=0.016).

### 3.2. Employed Treatments and Responses

No major differences in baseline patients' characteristics were found between DA-EPOCH-R, R-CHOP, and R-CHOEP groups (Table 2). Of the 93 patients, 41.9% were treated with DA-EPOCH-R, 35.5% with R-CHOP, and 22.5% with R-CHOEP. Cytoreduction with COP was used in 50% (46/92) of patients. Nine (9.7%) patients had to reduce treatment (from DA-EPOCH-R to R-CHOEP) due to lack of hospital beds or severe infection in a previous cycle. Still, DA-EPOCH-R patients who had their treatment intensified based on their previous white blood count and tolerance performed 74.5% (29/39), ranging from level 2 to 5 (Table 3). CNS prophylaxis was performed in 36/84 patients (42.9%), and 8 (22.2%) received high-dose methotrexate at the end of treatment.

Interim assessment was performed by PET-CT in approximately half of the patients (*n* = 45; 51.1%). Complete response was achieved in 35 patients (40%), with 14.8% of CR and 25% of CRu. PR was seen in 49/88 patients (55.7%), while PD, in 4/88 (4.5%).

Subsequent mediastinal RT was performed in 62.5% (55/88) of the patients, corresponding to 42% (16/38) of the DA-EPOCH-R, 75% (24/32) of R-CHOP, and 83.3% (15/18) of R-CHOEP (*p*=0.002) groups. Nineteen (36.5%) of 52 patients were in PR before the irradiation and 73.7% (14/19) of them converted to CR. Three patients with SD or PD received RT after the end of treatment (5.7%), and all experienced further progression. Pre-RT disease status could not be assessed in three individuals. The median RT dose was 36 Gy (range 25.2–40).

Final assessment by PET-CT was performed in 100% of DA-EPOCH-R, 95% of R-CHOEP, and 78.8% of R-CHOP patients. By Cheson criteria [19], CR, Cru, and stable/RR rates were 22.3%, 55.3%, and 22.3%, respectively. Within stable/RR group, 11 patients were later considered in CR (two after a new PET-CT scan) and nine after detecting no relapse during follow-up (five had positive, two had negative, and two had inconclusive scans). By Lugano criteria, metabolic CR, PR, and stable/refractory disease rates were 69.7%, 16.7%, and 13.6%, respectively.

CR rate was not associated with any clinic-pathological features tested: obesity (*p*=0.875), smoking (*p*=0.295), bulky disease (*p*=0.204), thrombosis (*p*=0.987), albumin (*p*=0.652), age (*p*=0.842), stage (*p*=0.645), LDH (*p*=0.697), or sex (*p*=0.097). As for treatment choice, no statistically significant difference in CR rate was also found (DA-EPOCH-R 74.3%, R-CHOP 76.7%, and R-CHOEP 82.3%, *p*=0.895).

Multivariable analysis involving sex, cytoreductive COP, RT, and interim response showed that only mediastinal irradiation (OR = 4.61 (95% CI: 1.28–18.8), *p*=0.022) and an interim CR/CRu (OR = 6.25 (95% CI: 1.56–33.89), *p*=0.017) were predictors of final CR.

### 3.3. Relapse, Overall Survival, and Progression-Free Survival

**Figure 2:**
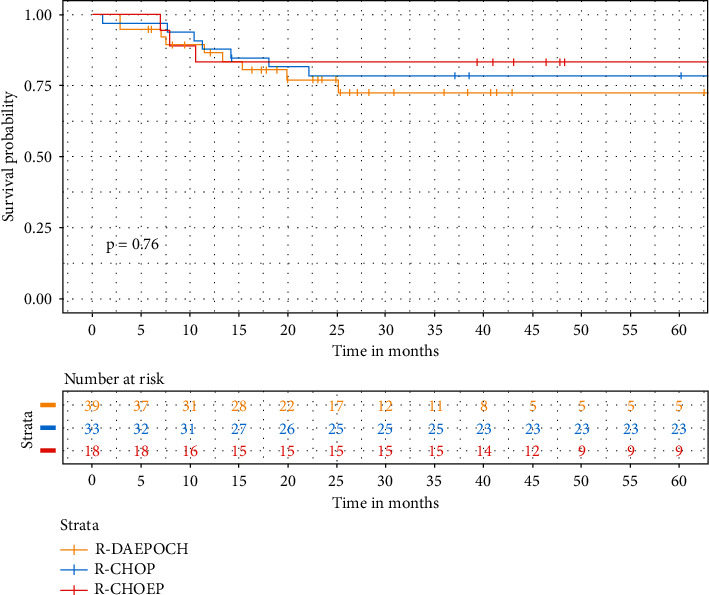
Survival probability by frontline therapy.

The median follow-up was 60.2 months. Estimated 5-year PFS and OS for the whole cohort were 77.2% (95% CI 68.9–86.6) and 77.4% (95% CI 68.9–87), respectively. PFS in DA-EPOCH-R, R-CHOP, and R-CHOEP groups was 72.6%, 78.6%, and 81%, and OS was 70.3%, 78.4%, and 81% (*p*=0.76), respectively, with no statistical difference (Figure 2). Analyses for PFS and OS rates according to revised (R-IPI) and age-adjusted (aaIPI) IPI identified that only the subgroup with zero risk factors tended to better results, although without statistical differences (PFS: *p*=0.18 and *p*=0.49, and OS: *p*=0.23 and *p*=0.53, respectively). Patients with 1 to 2 and 3 to 5 risk factors had similar PFS and OS. See figures a, b, c, and d in the supplementary appendix.

The 5-year CIR was 9.2% (95% CI: 4.2–16.4) with eight cases. Half of them (4/8 patients) had CNS involvement at the relapse. There was also no difference in CIR according to the regimens—DA-EPOCH-R 13.2%, R-CHOP 6%, and R-CHOEP 11.1% (*p*=0.71).

**Figure 3:**
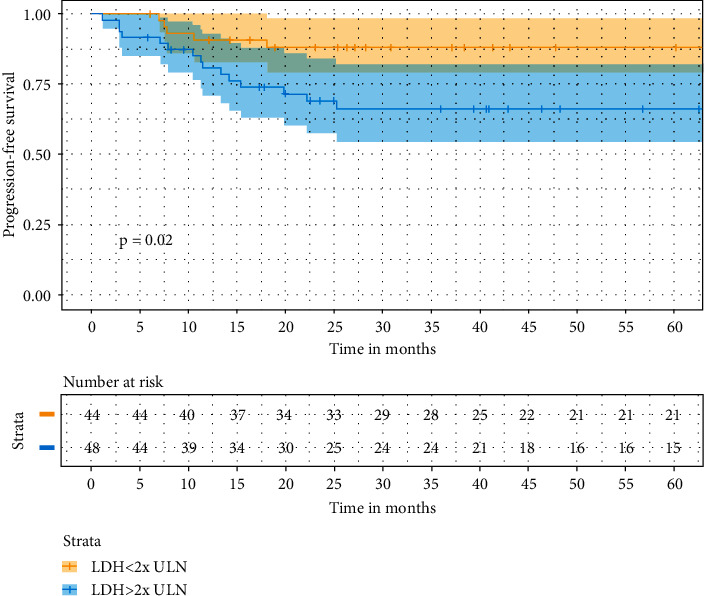
Kaplan–Meier curve for PFS according to the baseline LDH level.

UVA for survival demonstrated that only higher levels of LDH (PFS 88.2 vs. 66.3%) were associated with worse PFS (hazard ratio (HR) 3.115, (95% CI: 1.13–8.58), *p*=0.02) and OS (*p*=0.03) (Figure 2). No other clinic-pathological variables were associated with survival, noticeably thrombosis (HR = 1.3 (95% CI: 0.54–3.3), *p*=0.5) and extranodal disease (HR = 1.5 (95% CI: 0.6–3.9), *p*=0.4). No difference was seen in impact of RT on survival within all treatment arms (DA-R-EPOCH, *p*=0.5; R-CHOP, *p*=0.09; R-CHOEP, *p*=0.3). In a MVA including LDH and interim response, only LDH remained independently associated with PFS (Figure 3).

As we do not dispose of anti-PD1 monoclonal antibodies or other class of novel agents, IVAC (*n* = 6) and ICE (*n* = 2) were the main salvage regimens adopted and only two patients managed to proceed to autologous hematopoietic stem-cell transplantation. Median time to relapse was 9.2 months (range 6.9–25.2). Details of each outcome per each treatment arm are depicted in the flowchart in the supplementary appendix.

There were eight deaths in the DA-EPOCH-R arm, one before the 3rd cycle and one at the end of the initial treatment, both from septic shock. The remaining six died mainly due to PD in the subsequent lines (range 1 to 4). In the less intensive group, three patients died of infectious complications from febrile neutropenia before the interim assessment could be done (R-CHOP: 2; R-CHOEP: 1). Four died from progressive disease and four from septic shock during subsequent therapies (range 1 to 3). One patient died of gastric adenocarcinoma, while in CR from lymphoma, seven years after treatment with R-CHOP.

### 3.4. Adverse Events and Toxicities

Serious infectious adverse events were observed (Grade 4) only in the DA-EPOCH-R group (mainly pulmonary septic shocks associated with prolonged neutropenia; *n* = 4). One case of meningitis was also reported, as well as one case of subdural chronic hematoma. Grade 3 infectious events, mainly febrile neutropenia, were seen in all groups (DA-EPOCH-R: 11; R-CHOEP: 9; and R-CHOP: 4).

## 4. Discussion

While the introduction of rituximab greatly improved the outcomes of CHOP plus RT in PMBCL [7, 22], the same could not be said about further intensification of the chemotherapy itself, as attempted by several groups [7, 9, 23, 24]. Dunleavy et al. then showed the world remarkable results of a phase II trial with 67 patients submitted to a non-RT-based DA-EPOCH-R, giving this regimen a high scientific acceptance [13]. However, some later real-life evidences may suggest that, in some specific circumstances, DA-EPOCH-R might not be that better than R-CHOP, and also that the mediastinal RT might still have its role in the management of PMBCL [11, 25–28]. Despite all its statistical limitations and bias, our findings find a path with those studies, summarized in Table 4.

Although approximately one-third of our patients received R-CHOP as upfront treatment, a gradual replacement by DA-EPOCH-R could be observed, notably in the last five years. Nevertheless, in many cases, we were forced to reduce from DA-EPOCH-R to a less intensive R-CHOEP, due to the unavailability of hospital beds or infusion pumps. CHOEP in PMBCL is not new. In the Mabthera International Trial Group (MInT), 39 in 87 PMBCL patients were treated with CHOEP, some of them with rituximab [30]. Wang et al. also reported a Chinese experience with CHOEP in a small cohort of 29 patients, with a 5-year OS of 85% [31]. So, this regimen is acceptable for PMBCL according to literature, yet not deeply explored.

In parallel, real life showed that mediastinal RT is still employed in PMBCL, including in a sizable number of patients treated with DA-EPOCH-R, as shown by Giulino-Roth et al. (15%) and Zhou et al. (69%) [25,32]. Our work found that an excessive 45% of DA-EPOCH-R patients received RT and, although not consistent to current recommendations [3,33], this might be associated with the lack of consensus on the management of the residual mediastinal uptake at PET-CT scans. Besides, the unavailability of newer agents for refractory or relapsed disease in our setting may have affected the clinician's decision on RT referral and, consequently, our numbers when compared to literature. Besides, it is known that few patients with a positive end-of-treatment PET-CT will actually evolve to a progression or relapse, and that a serial PET imaging might obviate unnecessary RT after DA-EPOCH-R [14]. But this is not true for patients with frankly positive disease at the end of treatment (e.g., a Deauville 5 mediastinal uptake), where even additional RT seems to be insufficient [28]. According to our findings, RT also did not rescue patients with SD/PD (or Deauville 5 mediastinal uptake) either.

Several cohorts have attempted to find baseline characteristics associated with PFS in PMBCL. While the International Prognostic Index (IPI) seems to be suboptimal to prognosticate those patients, there is a general agreement on the extranodal disease and elevated LDH as adverse factors [28]. Here, only LDH was independently associated with PFS. And thrombosis was only marginally associated with survival, differently from Lekovic et al. [34].

Brazil offers universal and unified system as a public health management model. Global and free access to medical care is guaranteed by the 1988 Constitution, when the model was created. However, the supply of beds for the treatment for oncological diseases is still scarce. When present, they often end up destined for the treatment of complications related to chemotherapy in detriment of hospitalizations for continuous chemotherapy infusion. The adoption of a regimen that requires hospitalization or drug infusion devices may not be the most suitable for some environments such as ours, despite its good outcome and acceptance worldwide. For this reason, having a better understanding of the impact of what we have been doing for the last decade is important for us to trace treatment strategies for the future. For each choice, we must consider the clinical burden, not only related to treatment itself but to early and late developments. This is particularly true for PMBCL, since it may have a better prognosis in frontline setting when compared to DLBCL, but usually reserves a poor outcome after salvage therapy. However, overtreatment itself may lead to long-term consequences, especially in such a young group of patients. Furthermore, there is the economic impact of these strategies that could not be away of any debate in any health system, public or not. Recently, Yang et al. designed a study concerning the costs of the treatment of PMBCL in USA, with a population contemporary to our study, R-CHOP was the most recorded regimen as frontline therapy for PMBCL, and the cost of the treatment for a regular patient could range between 94,000 and 149,000 USA dollars [35]. Whittington et al. also tried to evaluate the long-term survival and cost-effectiveness of both chemotherapy and chimeric antigen receptor T-cell (CAR-T) therapy, with better outcome for the CAR-T arm, considering quality-adjusted life-years (QALY) at the end of the trial in 24 months [36].

We must highlight that our study has several limitations such as its retrospective feature, small number of patients enrolled over a long time of period of accrual, and a heterogeneous group regarding choice of treatment, which again changed according to practice over time, firstly with R-CHOP or R-CHOEP, and after 2013, DA-EPOCH-R. Because of those limitations, our results do not support any new relevant conclusions that might change the current state of the art in PMBCL. But they do confirm that conventional R-CHOP/CHOP-like ± RT is still a suitable regimen option in resource-limited settings.

## 5. Conclusion

In this cohort, we described the treatment patterns and outcomes of PMBCL in a resource-limited setting. In the face of increasing knowledge about the biology of cancer and innovative treatment strategies, we must still weigh the pros and cons before adopting new regimens in our midst. While an intensive—but costly—regimen has proven to be the most effective, it may not be the most suitable in some scenarios.

## Figures and Tables

**Table 1:** Baseline characteristics of total cohort (*n* = 93).

Age—median (range, IQR)	28 (15–74, 25–37)
Gender–% (*n*)	Female: 59.1% (55/93)
Prior smoking^**a**^	25.3% (22/87)
BMI (kg/m^2^)—median (range, IQR)	23.2 (15.4–40.1, 20.8–27.2)
Obesity, % (*n*)	7.6 (7)
Hemoglobin (g/dL)—median (range, IQR)	12.1 (3.8–15.8, 11.3–13.3)
Neutrophils (×10^9^/L)—median (range, IQR)	6.6 (1.8–16.6, 4.5–9.6)
Lymphocytes (×10^9^/L)—median (range, IQR)	1.1 (0.1–9.5, 0.6–1.6)
Monocytes (×10^9^/L)—median (range, IQR)	0.8 (0–2.8, 0.6–1.2)
LMR (lymphocyte-to-monocyte ratio)—median (range, IQR)	1.3 (0.15–8.6, 0.9–2)
Platelets (×10^9^/L)–—median (range, IQR)	361 (149–837, 290–440)
Albumin (g/dl)–—median (range, IQR)	3.9 (1.5–5.1, 3.5–4.3)
Abnormal lactate dehydrogenase (>ULN)	86.9%
Abnormal lactate dehydrogenase (>2x ULN)	52.2% (48/92)^**b**^
Staging method	PET, 84.7%
	CTs plus BM biopsy, 12.1%
	CTs without BM biopsy, 3.2%
Ann Arbor staging§	I (13, 14.3%)
	II (39, 42.8%)
	III (10, 11%)
	IV (29, 31.9%)
Contiguous extranodal disease	Pleura (61.5%)
	Pericardium (44.6%)
	Lungs (32.3%)
	Bones (30.8%)
	Heart (6.2%)
Noncontiguous extranodal disease	Kidneys (48%)
	Pancreas (32%)
	Lungs (28%)
	Adrenal (24%)
	Bones (16%)
Bulky disease, % (*n*)	92.1% (82/89)
B-symptoms % (*n*)	75.8% (69/91)^**c**^
ECOG, % (*n*)	0–2 : 91.2% (83/91)^**c**^
R-IPI	
Very good	9/87 (10.3%)
Good	46/87 (52.9%)
Poor	32/87 (36.8%)

IQR, interquartile range; BMI, body mass index; ULN, upper limit of normal; ECOG, Eastern Cooperative Study Group; IPI, International Prognostic Index; aaIPI, Age-adjusted International Prognostic Index; ^**a**^ missing data in 6 patients; ^**b**^ missing data for one patient; ^**c**^missing data for two patients.

**Table 2:** Comparison among baseline characteristics of the three treatment groups.

	DA-R-EPOCH (*n* = 39)	R-CHOP (*n* = 33)	R-CHOEP (*n* = 18)	*p*-value
Sex (female, %)	59	57.6	61.1	0.970
Age (mean)	30.3	36	30.1	0.073
LDH (mean)	3.29	2	2.44	0.035
LDH (>2ULN, %)	64.1	40.6	44.4	0.113
Albumin (mean)	3.7	4.1	4.03	0.023
B-symptoms (%)	74.4	80.6	72.2	0.753
LMR (mean)	1.48	2.12	1.44	0.110
LMR (<1.3, %)	53.8	36.4	52.9	0.291
Thrombosis (%)	38.5	32.3	41.2	0.794
Advanced stage (%)	53.8	25.8	44.4	0.06
Bulky disease (%)	92.3	90.3	93.8	0.912
Cytoreductive COP (%)	48.7	63.6	23.5	0.027
R-IPI				0.21
Very good	6/39 (7.7%)	4/29 (13.8%)	2/20 (10%)	
Good	22/39 (56.4%)	12/29 (41.4%)	12/20 (60%)	
Poor	14/39 (35.9%)	13/29 (44.8%)	6/20 (30%)	
aaIPI				0.22
Low	3 (7.7%)	4 (13.8%)	2/20 (10%)	
Low-intermediate	11 (28.2%)	8 (27.6%)	9 (45%)	
High-intermediate	19 (48.7%)	11 (37.9%)	7 (35%)	
High	6 (25.4%)	6 (20.6%)	2 (10%)	

**Table 3:** Proportion of patients who received dose escalation in DA-EPOCH-R group (*n* = 39).

Level	*n*	%
1	10	25.6
2	8	20.6
3	10	25.6
4	10	25.6
5	1	2.6

Dose escalation: levels 2 to 5, according to Dunleavy et al., NEJM, 2013.

**Table 4:** Real-world data on R-CHOP versus DA-EPOCH-R in PMBCL.

Study	*N*	Consolidation RT	Survival	*p*-value
Shah et al. [25]	R-CHOP (*n* = 56)	R-CHOP: 59%	R-CHOP: 2 y PFS = 76%	0.28
	DA-EPOCH-R (*n* = 76)	DA-EPOCH-R: 13%	DA-EPOCH-R 28: 2 y PFS = 85%	
Malenda et al. [26]	R-CHOP (*n* = 25)	R-CHOP: 100%	R-CHOP: 1 y PFS = 87%	0.2
	DA-EPOCH-R (*n* = 28)	DA-EPOCH-R: 59%	DA-EPOCH-R: 5 y PFS = 73.9%	
Chan et al. [11]	R‐CHOP (*n* = 41)	R-CHOP: 47%	R‐CHOP: 5 y PFS = 56%	0.02
	R‐CHOP + RT (*n* = 37)	DA-EPOCH-R: 6%	R‐CHOP + RT: 5 y PFS = 90%	
	DA‐EPOCH‐R (*n* = 46)		DA‐EPOCH‐R: 5 y PFS = 88.5%	
Eule et al. [29]	R-CHOP (*n* = 9)	100% in R-CHOP arm	3 y OS (both arms): 96%	N.R.
	DA-EPOCH-R (*n* = 18)			
Present study	R-CHOP (*n* = 33)	R-CHOP: 83%	R-CHOP: 5 y PFS = 79%	0.9
	R-CHOEP (*n* = 18)	R-CHOEP: 83%	R-CHOEP: 5 y PFS = 83%	
	DA-EPOCH-R (*n* = 39)	DA-EPOCH-R: 44%	DA-EPOCH-R: 5 y PFS = 75%	

## Data Availability

No publicly available data were used to support this study.

## Supplementary Materials

Supplementary Materials The supplementary material for this article contains page 1—detailed chemotherapy regimens for this paper; page 2—flowchart of patients included; and figures a-d—PFS and OS according to aaIPI and R-IPI.
