# Supplementary material for: Less Intensive Regimens May Still Be Suitable for the Initial Treatment of Primary Mediastinal B-Cell Lymphoma in Resource-Limited Settings
Source: J Oncol. 2022 Jun 6;2022:2099456. doi: 10.1155/2022/2099456 (PMC9192285; doi:10.1155/2022/2099456)

***Supplementary appendix***

| Cytoreductive Regimen ‘COP’ | | | | |
| --- | --- | --- | --- | --- |
| Cyclophosphamide | 300 mg/sqm | IV in 2h | OD | D1 |
| Vincristine | 1 mg/sqm (capped at 2mg) | IV | OD | D1 |
| Prednisone | 60 mg/sqm | PO | OD | D1 to D7 |

| R-CHOP | | | |
| --- | --- | --- | --- |
| Rituximab | 375 mg/sqm | IV | D1 |
| Cyclophosphamide | 750 mg/sqm | IV | D1 |
| Vincristine | 1.4 mg/sqm (capped at 2mg) | IV | D1 |
| Doxorubicin | 50 mg/sqm | IV | D1 |
| Prednisone | 100 mg | PO | D1 to D5 |
| MTX 15 mg  Dexamethasone 2 mg | | IT | Each cycle |
| R-CHOEP | | | |
| Etoposide | 100 mg/sqm | IV | D1 to D3 |
| *Plus CHOP above mentioned* | | | |

R-CHOP and R-CHOEP * Reduced R-CHOEP: Etoposide 100mg/sqm IV on D1 only

*Flowchart of patients analyzed*


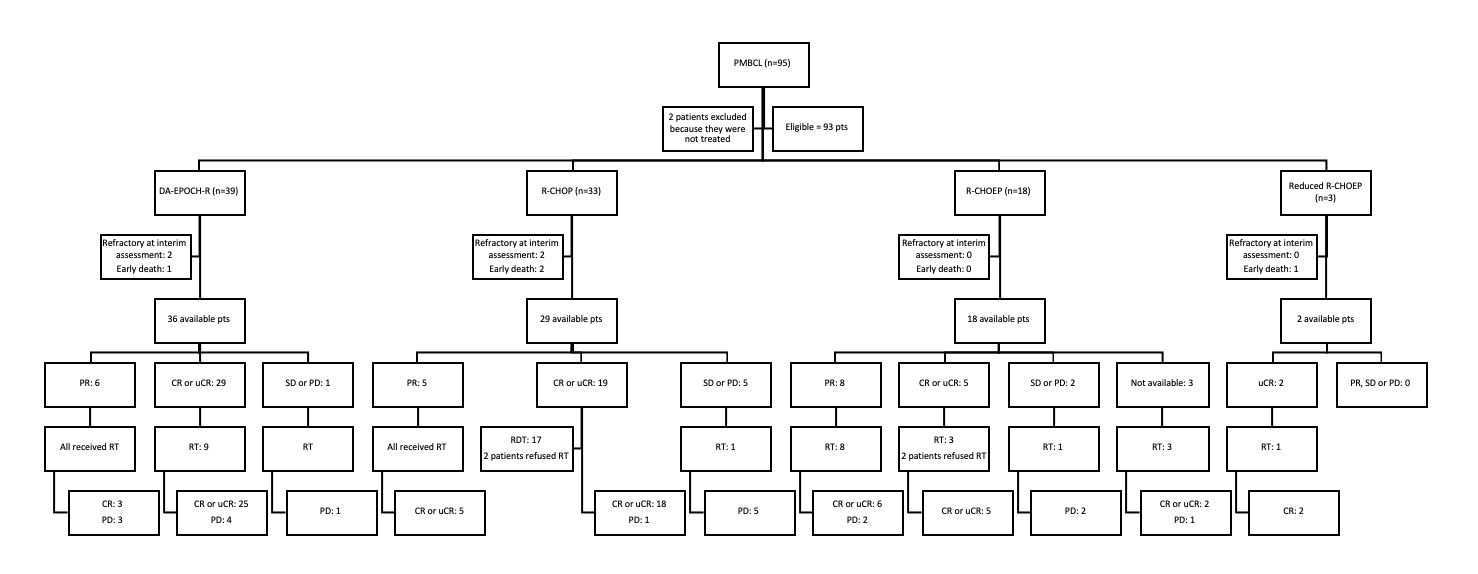


*Figure a. OS according to age-adjusted International Prognostic Index (aaIPI)*


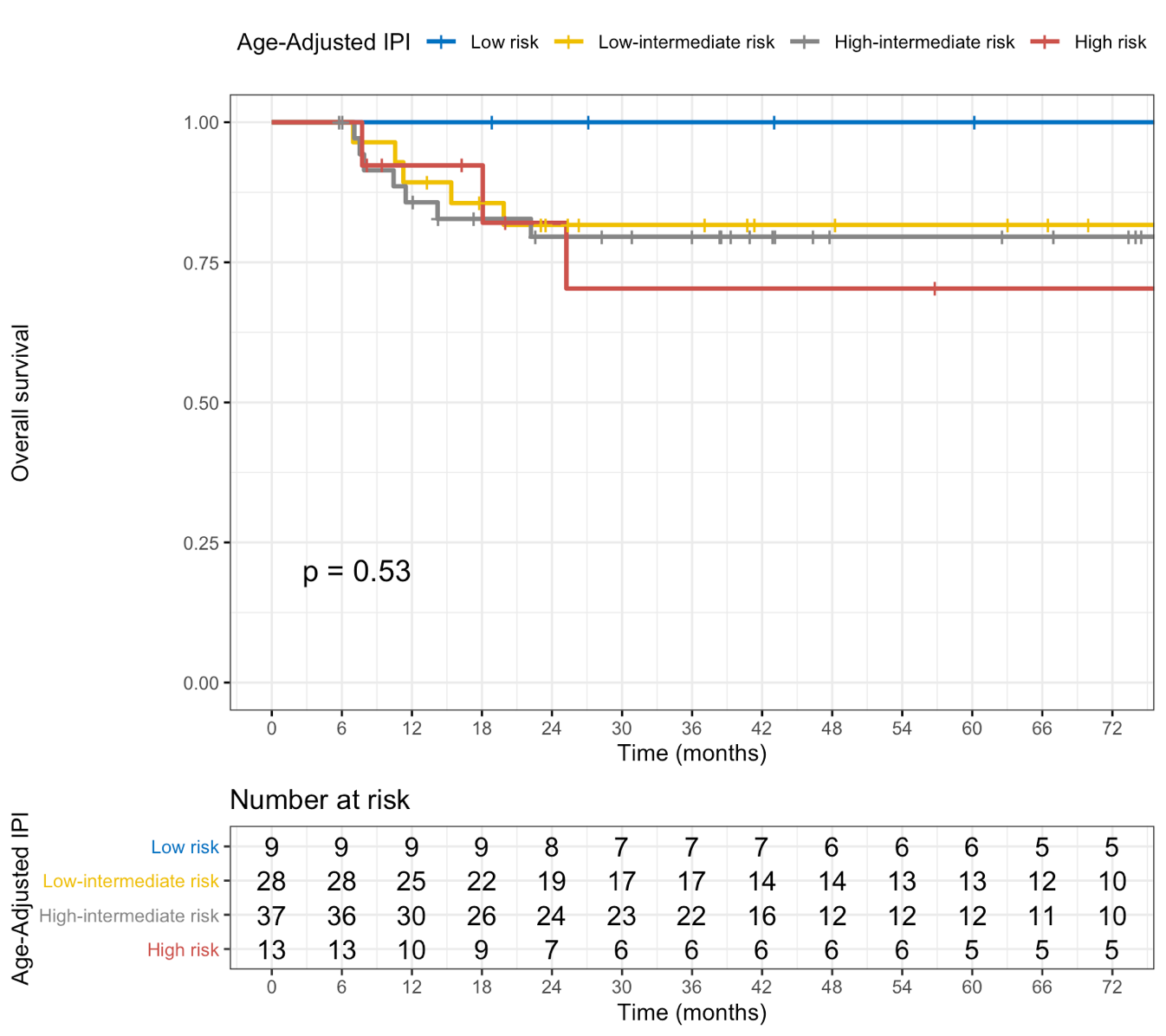


*Figure b. PFS according to age-adjusted International Prognostic Index (aaIPI)*


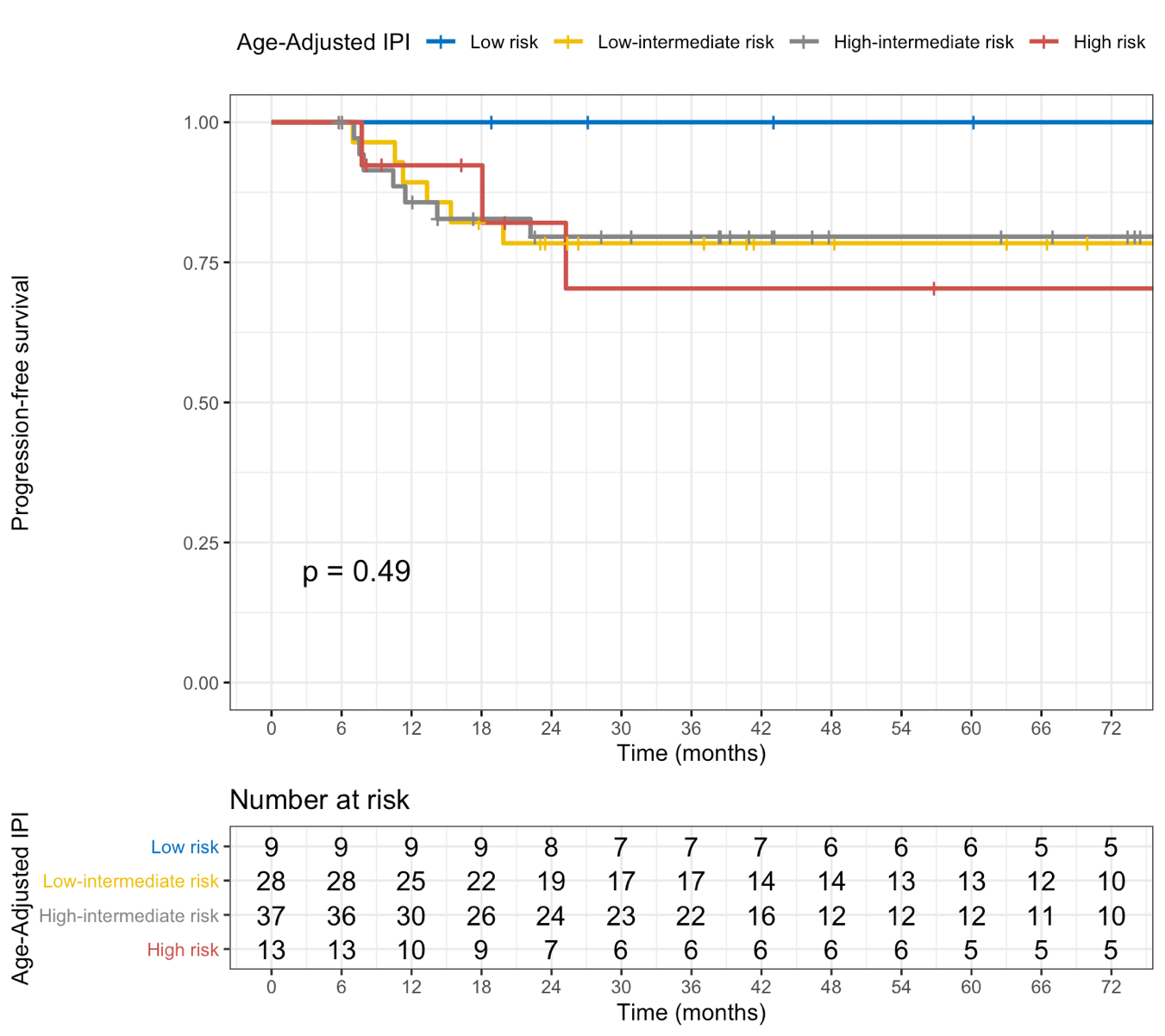


*Figure c. OS according to revised International Prognostic Index (R-IPI)*
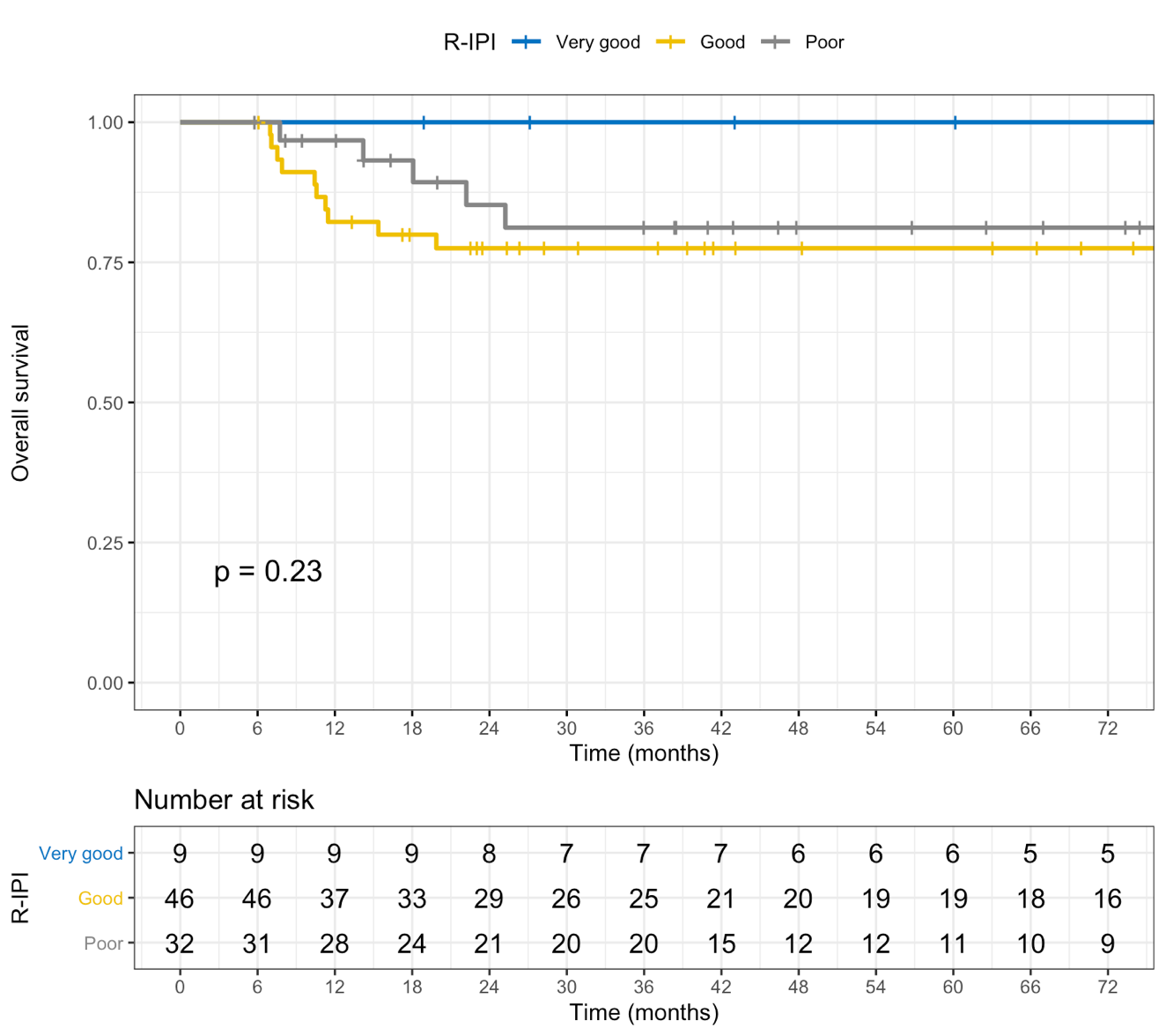


*Figure d. PFS according to revised International Prognostic Index (R-IPI)*
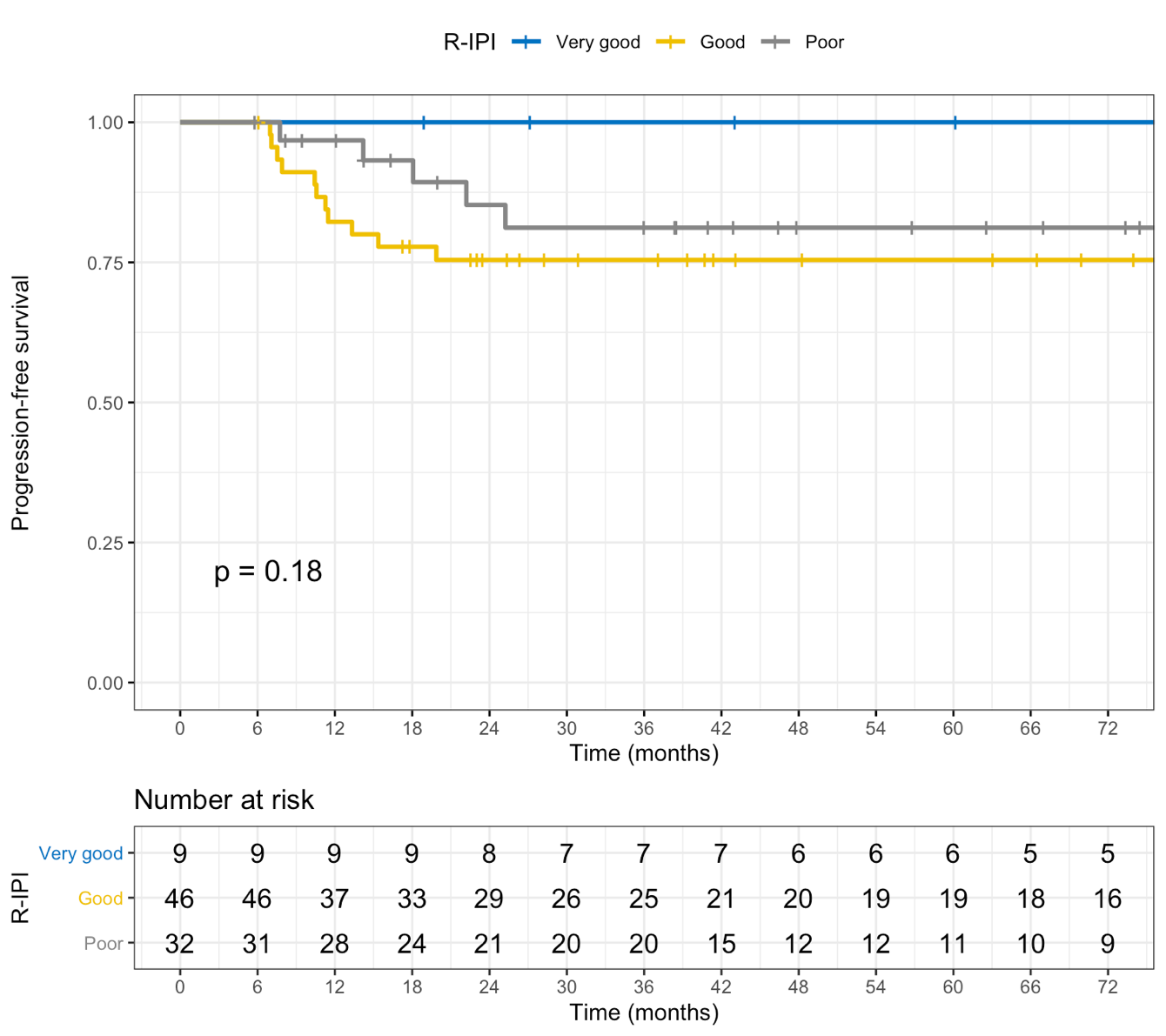

Supplement: Supplementary Materials — The supplementary material for this article contains page 1—detailed chemotherapy regimens for this paper; page 2—flowchart of patients included; and figures a-d—PFS and OS according to aaIPI and R-IPI. [file 2099456.f1.docx]
